# Inflammatory Linear Verrucous Epidermal Nevus (ILVEN): A Scarce Cognate with a Neoplasia

**DOI:** 10.1007/s13193-024-02051-4

**Published:** 2024-08-03

**Authors:** Jayavarmaa R, Gaurav Das

**Affiliations:** Department of Surgical Oncology, Dr. B. Borooah Cancer Institute, Guwahati, Assam India

**Keywords:** Inflammatory linear verrucous epidermal nevus, Blaschko’s lines, Trichoepithelioma, Surgical resection, V–Y Advancement flap

## Abstract

Inflammatory linear verrucous epidermal nevus (ILVEN) is a rare type of cutaneous nevus that has female predilection which occurs at birth or infancy and evolves along Blaschko’s lines, perceived unilaterally, over the buttocks, legs, and arms. In this case report, ILVEN was associated with multiple ulcero-proliferative skin lesions over the dorsum of the left foot, which on biopsy were proven to be malignant adnexal tumors. A 51-year-old female presented with linear, scaly, and verrucous skin lesions on her left buttock, thigh, and leg unilaterally along Blaschko’s lines after birth. Post-operative biopsy taken from the dorsum of the foot reveals trichoepithelioma. Biopsy taken from the back of the thigh and leg reveals inflammatory linear verrucous epidermal nevus. Single-stage surgical resection of both lesions, performed with primary closure of all the areas, except the gluteal region which is closed by V–Y advancement flap. The outcome was satisfactory for the patient after surgical resection and reconstruction. No recurrence was detected during the follow-up visits. ILVEN is an uncommon type of hyperplastic cutaneous disease. ILVEN perchance associated with malignancy. Henceforth, it has to be considered during the evaluation of similar lesions for optimal treatment intervention.

## Introduction

Inflammatory linear verrucous epidermal nevus (ILVEN) is an erythematous, linear, and pruritic scaly lesion with psoriasiform papules. Along Blaschko’s lines, the papules merge to form plaques, which are evident unilaterally at the lower limb [1]. In this case report, ILVEN was associated with multiple ulcero-proliferative skin lesions over the dorsum of the left foot, which on biopsy were proven to be malignant adnexal tumors.

## Case Presentation

**Fig. 1:**
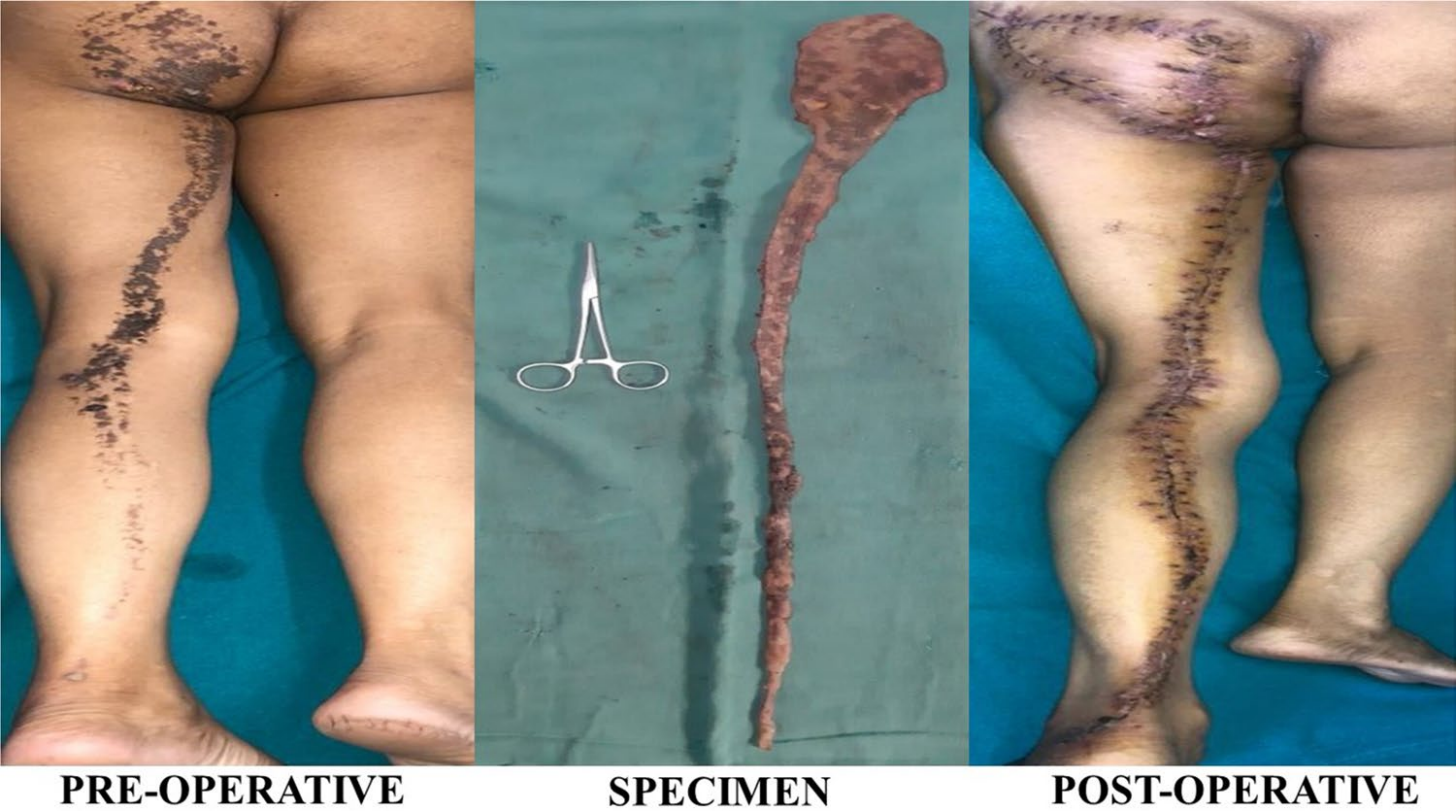
Inflammatory linear verrucous epidermal nevus

**Fig. 2:**
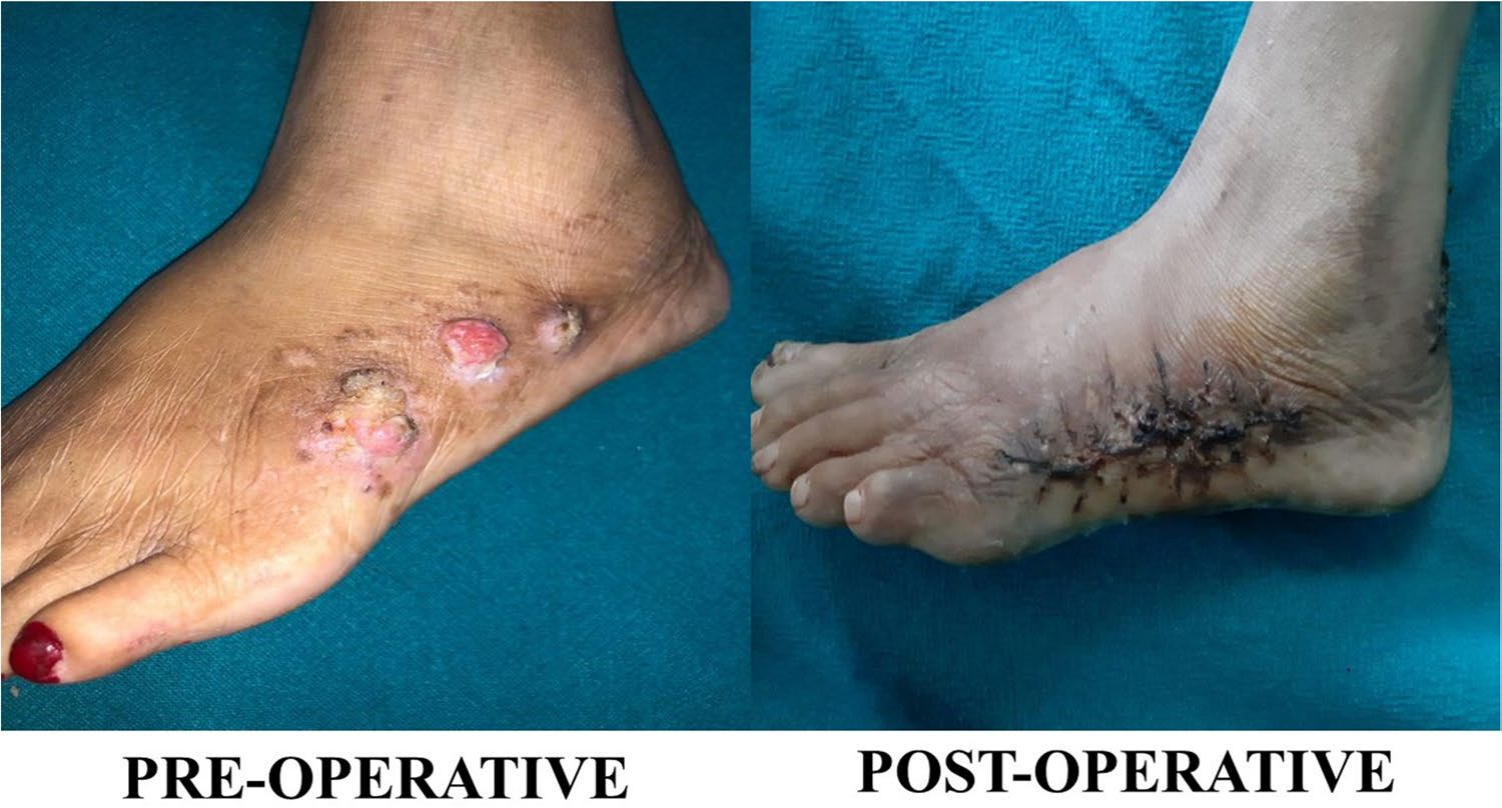
Trichoepithelioma

A 51-year-old female presented with linear, scaly, and verrucous skin lesions on her left buttock, thigh, and leg unilaterally along Blaschko’s lines after birth (Figs. 1 and 2).

A pre-operative biopsy taken from the dorsum of the foot lesion revealed invasive malignant adnexal carcinoma suggestive of eccrine porocarcinoma. A biopsy taken from the back of the thigh and leg lesion revealed an inflammatory, linear, verrucous, epidermal nevus.

Single-stage surgical resection of both lesions is performed with primary closure of all the areas except the gluteal region, which is closed by a V–Y advancement flap. The outcome was satisfactory for the patient after surgical resection and reconstruction. No recurrence was detected during the follow-up visits.

**Fig. 3:**
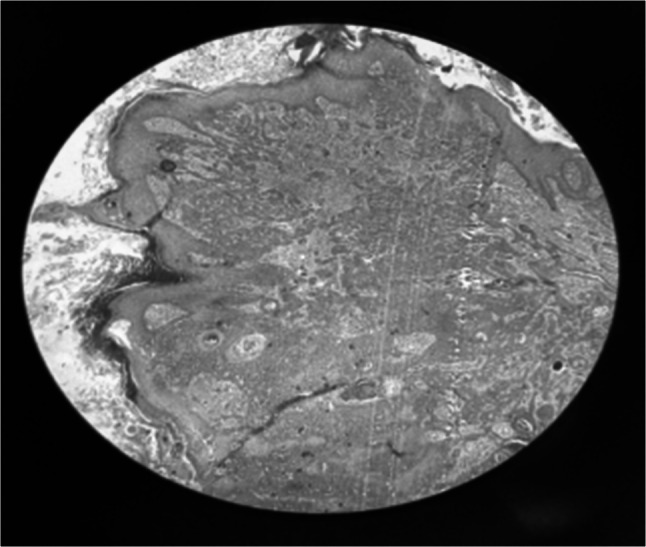
Biopsy taken from the dorsum of the foot showing hyperplastic lining epithelium with underlying sheets and nests of cells

**Fig. 4:**
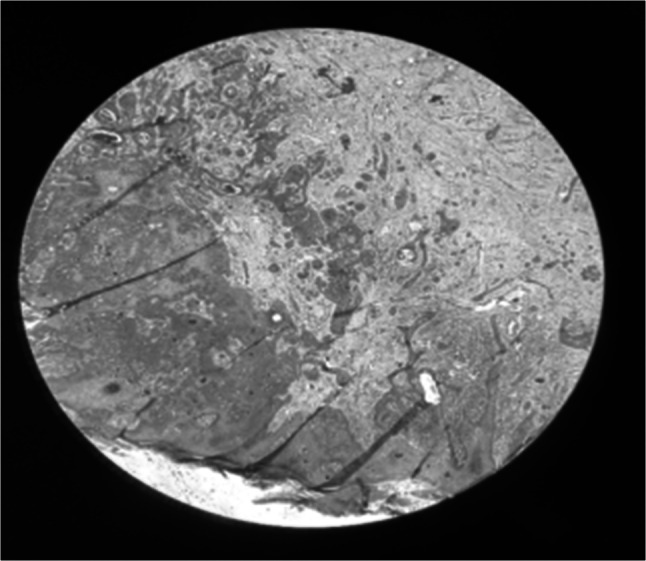
Biopsy taken from the back of the thigh and leg showing hyperplastic and hyperkeratotic lining

A post-operative biopsy taken from the dorsum of the foot showed hyperplastic lining epithelium with underlying sheets and nests of cells showing minimal atypia, no mitosis, and multiple horn cysts, suggestive of trichoepithelioma (Fig. 3). Biopsy taken from the back of the thigh and leg showed hyperplastic and hyperkeratotic lining, revealing inflammatory linear verrucous epidermal nevus (Fig. 4).

## Discussion

Inflammatory linear verrucous epidermal nevus (ILVEN) was introduced into the literature in 1971 by Mehregan and Altman [1] and defined in 1985 by Morag and Metzker [2]. It is an anomalous type of epidermal nevus that occurs at birth or infancy and evolves along Blaschko’s lines [3]. It has female predilection and is perceived unilaterally over the buttocks, legs, and arms [4, 5]. ILVEN clinically gives the impression of linear, erythematous plaque with elevated pruritus [6].

ILVEN is distinguished among the epidermal nevus under the keratinocyctic variety, and relatively 6% of epidermal nevus constitutes ILVEN [7]. The prevalence of ILVEN is estimated in 1:1000 live births and is rarely associated with oral mucosal lesions and skeletal and central nervous system abnormalities [8]. ILVEN pathogenicity involves environmental and genetic factors. The genesis of ILVEN has been traced by heterozygous somatic mutation of GJA1 [9].

Clinical and histological features elicit a resemblance with other dermal hyperplastic disorders; hence, accurate diagnosis is challenging. ILVEN histopathologically signifies orthokeratotic hyperkeratosis overlying the alternating areas of demarcated hypergranulosis and overlying parakeratosis with hypogranulosis. A perivascular inflammatory infiltrate composed of histiocytes and lymphocytes, extending over the irregular hyperplastic epidermis, is seen. Exocytosis, spongiosis, and the formation of microabscesses are seen with psoriasiform epidermal hyperplasia. Acanthosis, psoriasiform, and inflammatory cells are seen [10, 11].

Treatment response is dependent on the severity of the lesion. Conservatively, topical administration of retinols such as calcipotriol [12], acitretin [10], etanercept [13], a combination of fluocinonide with tacrolimus [14], and corticosteroids [15] is efficacious. Triamcinolone acetonide intralesional injections are used as a palliative method [16].

Pulsed C02 laser ablation [3], cryotherapy [17], and surgical excision [18] are done as a treatment for ILVEN. Full-thickness skin excision followed by skin grafting is very efficient for treating ILVEN [19]. Laser therapy has proven to be more efficient on comparison to surgery, as it is less invasive and re-epithelization is faster [20–22]. These treatment modalities provide esthetic excellence.

Twenty cases had been documented in the literature to the best of our knowledge [4, 5, 20, 23–39]. In this case, there was a diagnostic dilemma between benign and malignant lesions, as the pre-operative biopsy showed ILVEN associated with eccrine porocarcinoma (malignant adnexal tumor). Hence, before the surgery, a definitive elucidation regarding the associated malignancy was mentioned. Although, post-operative histopathology report stated basal cell carcinoma and trichoepithelioma, as differential diagnosis. Decisively, trichoepithelioma was the final diagnosis, following immuno histochemistry. Henceforth, these types of skin lesions are always associated with challenges in diagnosis and may be associated with cutaneous malignancy. Periodically, this may create apprehension to patients and their caretakers which has to be handled carefully in explaining treatment modalities, in addition to the possibility of change in histopathological diagnosis after surgery.

## Conclusion

ILVEN is an uncommon type of hyperplastic cutaneous disease. ILVEN perchance is associated with malignancy. Henceforth, it has to be considered during the evaluation of similar lesions for optimal treatment intervention.

## Data Availability

All the data has been included in the case report itself.
